# Oleoylethanolamide‐induced anorexia in rats is associated with locomotor impairment

**DOI:** 10.14814/phy2.13517

**Published:** 2018-02-01

**Authors:** Shahana Fedele, Myrtha Arnold, Jean‐Philippe Krieger, Bernd Wolfstädter, Urs Meyer, Wolfgang Langhans, Abdelhak Mansouri

**Affiliations:** ^1^ Physiology and Behavior Laboratory ETH Zurich Schwerzenbach Switzerland; ^2^ Laboratorium für Organische Chemie ETH Zurich Zürich Switzerland; ^3^ Laboratory of Translational Nutrition Biology ETH Zurich Schwerzenbach Switzerland; ^4^ Institute of Pharmacology and Toxicology University of Zurich‐Vetsuisse Zurich Switzerland; ^5^ Neuroscience Center Zurich University of Zurich and ETH Zurich Zurich Switzerland

**Keywords:** Behavior, food intake, intestinal innervation, locomotor activity, side effects

## Abstract

The endogenous peroxisome proliferator‐activated receptor alpha (PPAR‐*α*) agonist Oleoylethanolamide (OEA) inhibits eating in rodents, mainly by delaying the onset of meals. The underlying mechanisms of OEA‐induced anorexia, however, remain unclear. Animals treated with high OEA doses were shown to display signs of discomfort and impaired locomotion. Therefore, we first examined whether the impaired locomotion may contribute to OEA's anorectic effect. Second, it is controversial whether abdominal vagal afferents are necessary for OEA's anorectic effect. Thus, we explored alternative peripheral neural pathways mediating IP OEA's anorectic effect by performing a celiac‐superior mesenteric ganglionectomy (CGX) or a subdiaphragmatic vagal deafferentation (SDA) alone or in combination. Exogenously administered OEA at a commonly used dose (10 mg/kg BW, IP) concurrently reduced food intake and compromised locomotor activity. Attempts to dissociate both phenomena using the dopamine D2/D3 receptor agonist Quinpirole (1 mg/kg BW, SC) failed because Quinpirole antagonized both, OEA‐induced locomotor impairment and delay in eating onset. CGX attenuated the prolongation of the latency to eat by IP OEA, but neither SDA nor CGX prevented IP OEA‐induced locomotor impairment. Our results indicate that IP OEA's anorectic effect may be secondary to impaired locomotion rather than due to physiological satiety. They further confirm that vagal afferents do not mediate exogenous OEA's anorectic effects, but suggest a role for spinal afferents in addition to an alternative, nonneuronal signaling route.

## Introduction

To control energy intake and energy expenditure, complex brain circuits integrate peripheral homeostatic signals. N‐acylethanolamines, lipid‐derived molecules with an ethanolamide moiety, supposedly provide such signals (DiPatrizio 2016). Oleoylethanolamide (OEA) is a fatty acid ethanolamide synthesized from oleic acid, mainly in the intestine in response to a fatty meal (Peterson et al. 2006; Fu et al. 2007, 2008; Schwartz et al. 2008). OEA has been shown to potently reduce food intake in rodents after peripheral administration (Rodriguez de Fonseca et al. 2001; Fu et al. 2003; Gaetani et al. 2003) and was therefore even suggested as a possible therapeutic agent to fight obesity (Romano et al. 2014). In freely eating rats, intraperitoneal (IP) or oral administration of OEA reduces food intake mainly by prolonging the latency to eat without affecting meal size or postmeal interval (Rodriguez de Fonseca et al. 2001; Gaetani et al. 2003; Azari et al. 2014). The results from pharmacological and transgenic studies suggest that OEA's anorectic action is mediated by the peroxisome proliferator‐activated receptor alpha (PPAR*α*) (Fu et al. 2003; Lo Verme et al. 2005; Peterson et al. 2006; Azari et al. 2013), but the exact mechanism through which OEA inhibits eating is still uncertain. Rodriguez de Fonseca et al. (2001) argued for an involvement of sensory fibers of the abdominal vagus, but recent studies from our laboratory (Azari et al. 2014) challenge this concept. OEA still potently reduced food intake in rats after subdiaphragmatic vagal deafferentation (SDA), a surgical procedure that eliminates all abdominal vagal afferents, while sparing half of the efferents.

Several studies indicate that OEA does not induce visceral illness or aversion (Proulx et al. 2005; Rodriguez de Fonseca et al. 2001), but a high dose of IP OEA (20 mg/kg) was shown to impair locomotor activity in rats, a finding that was judged negligible in relation to the reduction in food intake (Rodriguez de Fonseca et al. 2001). Years later, Wang and colleagues showed that IP administered OEA in mice (25 mg/kg) produced pronounced nociceptive behaviors (Wang et al. 2005), and others noted a decrease in ambulation and increase in inactivity time in rats following IP OEA (20 mg/kg) administration (Proulx et al. 2005). Similar observations in early stages of our work prompted us to investigate whether this locomotor impairment may be relevant for OEA's anorectic effect. We attempted to dissociate the latency to eat from the locomotor impairment to assess the OEA‐induced anorexia without confounding elements by first delaying food access and second by pharmacologically rescuing locomotion. In this respect, the dopamine D2/D3 agonist Quinpirole has been shown to increase locomotor activity in doses above 0.5 mg/kg and after 60 min upon injection (Eilam and Szechtman 1989), making it a pharmacologic candidate for restoring baseline locomotion.

Also, the findings that intracerebroventricular administration of OEA has no effect on food intake (Rodriguez de Fonseca et al. 2001) and that vagal afferents are not necessary for the anorectic effect of OEA (Azari et al. 2014) indicated an alternative peripheral, vagus nerve‐independent, mechanism of action. We therefore examined whether spinal afferents (whose cell bodies are localized in the dorsal root ganglia (DRGs)) may be involved in conveying the OEA‐derived anorectic signal. By surgical removal of the majority of the spinal afferent fibers connecting the gastrointestinal tract with the brain (celiac superior mesenteric ganglionectomy, CGX), and by combining the CGX procedure with the SDA, we examined the necessity of gastrointestinal afferent nerves (spinal and vagal) for IP administered OEA‐induced anorexia. Our results provide evidence for a causal relation between locomotor impairment and observed reduction in food intake and suggest an alternative, nonneuronal, route of action for OEA.

## Methods

### Animals and housing

Male Sprague‐Dawley rats (Charles River), weighing 160–180 g upon arrival, were housed individually in a climate‐controlled room (22 ± 2°C and 55 ± 5% relative humidity) under a 12/12 h dark/light cycle with ad libitum access to water and standard chow (Kliba 3436). All procedures were approved by the Veterinary Office of the Canton of Zurich.

### Drugs

Oleoylethanolamide (OEA) (Cayman n.90265) was dissolved in sterile saline/polyethylene glycol/tween 80 (90/5/5 v/v, 2 mL/kg (BW) and infused at the dose of 10 mg/kg BW through the IP catheter (1 min/mL) at the beginning of food intake or locomotor activity recording, unless otherwise stated. Quinpirole (Sigma Aldrich n. Q102) was dissolved in saline and subcutaneously (SC) injected at a dose of 1 mg/kg BW (1 mL/kg) 1 h prior to OEA administration.

### Catheter assembly

The catheters were in‐house handmade as described earlier (Azari et al. 2014). Briefly, the catheters consisted of silicone tubing [Dow Corning,; inner diameter (ID) × outer diameter (OD), 0.51 × 0.91 mm] connected to a polished L‐shaped 22‐gauge needle (Sterican, B. Braun). The connections between tubing and needles were shielded with 3‐mm (ID × OD, 0.76 × 1.65 mm) and 2.2 cm (ID × OD, 1.02 × 2.18 mm) long pieces of silicone tubing as inner and outer layers, respectively.

### Surgery preparations

All surgeries were performed under aseptic conditions. Prior to surgery, rats received a SC injection of antibiotics (20 mg/kg BW of sulfadoxine, Borgal 24%; Intervet/Shering‐Plough) for infection prophylaxis. An IP injection of atropine (0.05 mg/kg BW; Sintetica) was given before rats were anesthetized by isoflurane. Postoperative care consisted of antibiotics (1 day) and analgesic treatment (2 days).

#### IP catheter implantation

The proximal end of the catheter was led subcutaneously from the neck to a midline incision in the abdomen and inserted in the abdominal cavity through a puncture hole. Intraperitoneal catheters ended in the peritoneal cavity and were anchored on the left side of the abdominal wall with Histoacryl^®^ glue (B. Braun Medical).

#### Subdiaphragmatic vagal deafferentation

The Subdiaphragmatic vagal deafferentation (SDA) surgery was adapted from the method established by Norgren and Smith (1994) as described in detail previously (Arnold et al. 2006; Rüttimann et al. 2009). Briefly, it consisted of a left‐side intracranial vagal rhizotomy and a transection of the dorsal (right) subdiaphragmatic trunk of the vagus nerve. The SDA results in a complete disconnection of the abdominal afferents, while sparing half of the abdominal vagal efferents. SDA completeness was verified using an established functional test ascertaining the lack of cholecystokinin (CCK) satiation that depends on intact abdominal vagal afferent fibers (Smith and Gibbs 1985). Based on this criterion, we excluded two animals from the final analysis.

#### Celiac superior mesenteric ganglionectomy

As described by Sclafani et al. (2003), a 4–5 cm incision on the left side of the midline was performed and the left kidney, head of the spleen and pancreas were identified. Organs were gently retracted, overlying connective tissue was removed by blunt dissection, and the superior celiac ganglion was exposed. Localized between the descending aorta, celiac artery, and mesenteric artery, it assumes a star‐shaped structure with radiating processes. The radiations were identified and carefully cut to allow ganglion removal. Any additional neural tissue along the aorta, celiac artery and cranial mesenteric artery in the considered area was also transected. Celiac superior mesenteric ganglionectomy (CGX) completeness was confirmed by measuring the norepinephrine (NE) levels in intestinal tissues, and no animals were excluded. Some animals underwent the combination of SDA and CGX, without displaying any complication. Sham surgery consisted of exposing the vagal rootlets and dorsal subdiaphragmatic vagus similarly to the SDA procedure, but without manipulating them, combined with the exposure without further alteration of the celiac‐mesenteric ganglion.

### Food intake measurement and meal pattern analysis

Grounded chow (Kliba 3433) was available through a niche from feeding containers placed on scales (XS4001S; Mettler‐Toledo) connected to a computer with custom‐designed software (LabX meal analyzer 1.4, Mettler‐Toledo) that continuously recorded food intake. Meals were defined as food removals ≥0.3 g separated by ≥15 min of noneating as described previously by us and others (Farley et al. 2003; Azari et al. 2013; Punjabi et al. 2014) The satiety ratio was defined as the ratio between the first postmeal interval (min) and the first meal size (g). For food intake experiments, rats were food deprived for 1 hour and re‐fed at dark onset.

### Two bottles conditioned taste avoidance test:

Animals were adapted for 6 days to a daily water deprivation schedule with 2 h water access at the end of the light phase. Water was presented in two different bottles whose location was randomized during the adaptation period. Animals had ad libitum access to food. On the conditioning day, animals were offered for 30 min a 0.125% saccharin solution prior to infusion of NaCl (control), LiCl (60 mg/kg/9.4 mL in water) or OEA (10 mg/kg). Water was then offered for additional 90 min. After one intervening day, on which water was again presented for 2 h, on the test day, one bottle of water and one bottle of the saccharin solution were offered at random locations and 30 min intakes were recorded.

### Open field test

The test was carried out in two identical square arenas (80 × 80 cm) surrounded by walls 50 cm high, and a digital camera was mounted directly above the two arenas. The open field apparatus was made of grey Plexiglas and was located in a testing room under diffused lighting (30 lux as measured in the center of the arenas). Images were transmitted to a PC running the EthoVision (Noldus IT) tracking system. All tests were carried out during the dark phase and lasted 30 min. Experimenters who were blinded to the treatments analyzed the recorded video tapes for abdominals writhes: an arching of back, extension of hind limbs, and contraction of abdominal musculature and lateral torsions: an unnatural lateral displacement of the body weight on two limbs.

### Tissue collection and gene expression

Animals received an IP infusion of pentobarbital‐Na (100 mg/kg; Cantonal Pharmacy Zurich) 60 min after OEA or vehicle were infused, and the nodose ganglia (NG), DRGs (T5‐T11), and duodenum were promptly collected, frozen in liquid nitrogen and stored at −80°C. NGs and DRGs from the same animal were pooled before RNA was extracted using Trizol (Life Technologies). RT‐quantitative PCR (qPCR) was performed using SybR Green on a OneStep Plus instrument (Applied Biosystems), and results were analyzed, using the 2ddCt method. The following qPCR primers were used: *cfos*: F‐ AGCATGGGCTCCCCTGTCA, R‐ GAGACCAGAGTGGGCTGCA and Neuron‐specific enolase (*Eno2*): F‐ GGGGCACTCTACCAGGACTT, R‐ GGTCGAATGGGTCTTCAATG.

### Statistical analysis

**Figure 1:**
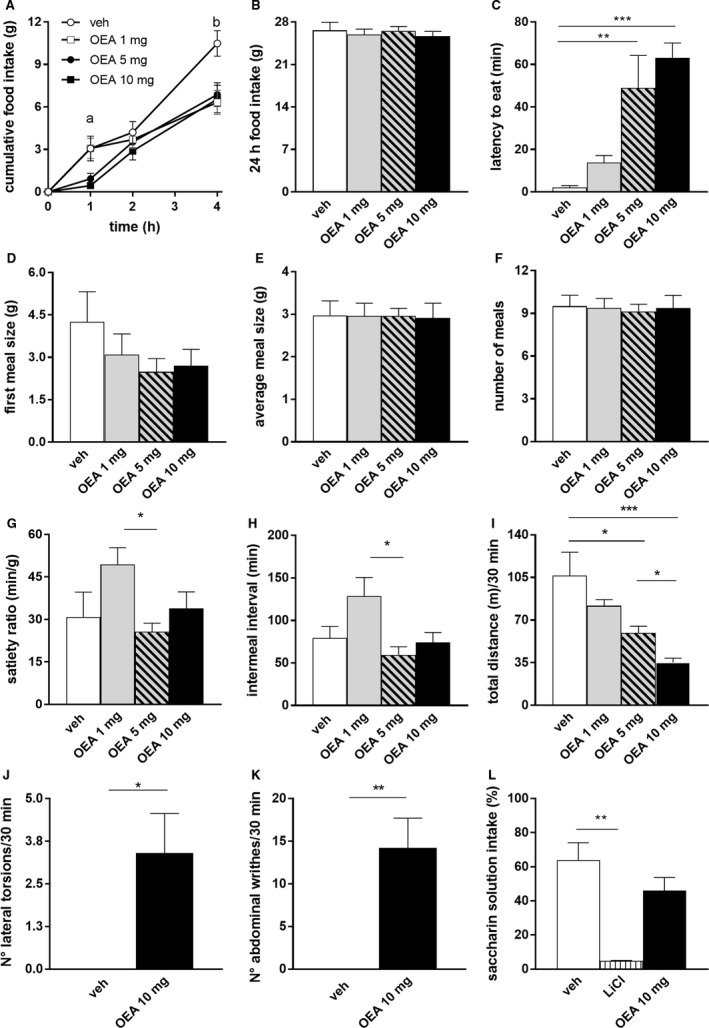
OEA infusion reduced short‐term food intake, prolonged latency to eat and impaired locomotor activity without inducing avoidance. (A–H) food intake and meal pattern analysis after OEA (1, 5, 10 mg/kg BW) or veh administration (*n* = 8). Short‐term (4 h) food intake (A), 2‐way ANOVA, group *F*(3,28) = 4.695, *P* < 0.01, time *F*(3,84) = 138.2, *P* < 0.01, group × time *F*(9,84) = 3.509 *P* < 0.01 followed by Tukey's multiple comparison test (*a* = veh vs. 5, 10 mg OEA *P* < 0.05; *b* = veh vs. 1, 5, 10 mg OEA,* P* < 0.001). 24 h food intake (B), ANOVA ns. Latency to eat (C), Kruskal–Wallis *P* < 0.05, followed by Dunn's multiple comparison test. First meal size (D), average meal size (E) and number of meals (F), ANOVA, ns. Satiety ratio (G) and intermeal interval (between first and second meal) (H), ANOVA, respectively, ns and *P* < 0.05, followed by Tukey's multiple comparison test. (I–K) open field test recordings after OEA (1, 5, 10 mg/kg BW) or veh administration (*n* = 5). Total distance moved in 30 min (I), ANOVA,* P* < 0.01 followed by Tukey's multiple comparison test. OEA‐induced motor behaviors: number of lateral torsions (J) and number of abdominal writhes (K), Student's *t*‐test, *P* < 0.05 and *P* < 0.01, respectively. (L) Saccharin preference ratio (saccharin solution intake in % of total fluid) after veh, (*n* = 6) LiCl (*n* = 3) or OEA (10 mg/kg, *n* = 8). 1‐way ANOVA,* P* < 0.05, followed by Tukey's multiple comparison test. Results are presented as means ± SEM. veh = vehicle, ns = statistically not significant, *P < 0.05, **P < 0.01, ***P < 0.001.

**Figure 2:**
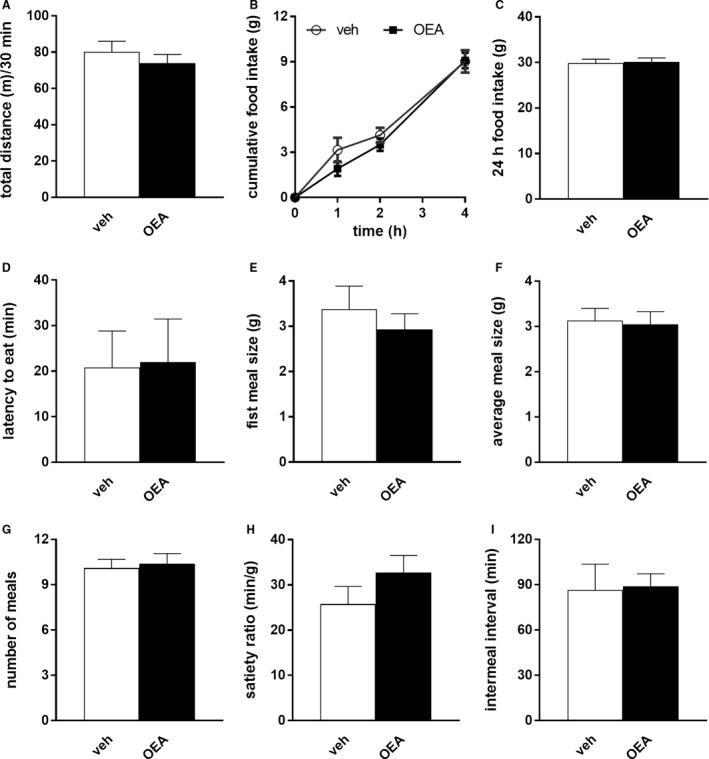
OEA did not affect locomotor activity or food intake when measurements started 1‐h postadministration. (A) locomotor activity in an open field test 1‐h post‐OEA (10 mg/kg BW) or veh administration (*n* = 10), Student *t*‐test, ns. (B–I) food intake and meal pattern analysis 1‐h post‐OEA (10 mg/kg BW) or veh administration (*n* = 10). Short‐term (4 h) food intake (B), 2‐way ANOVA, group *F*(1,9) = 1.497 ns, time *F*(3,27) = 124.9 *P* < 0.01, group × time *F*(3,27) = 1.589 ns. 24 h food intake (C), Student *t*‐test, ns. Latency to eat (D), Mann–Whitney *U* test, ns. First meal size (E), average meal size (F), number of meals (G) satiety ratio (H), intermeal interval (I), Student *t*‐tests, all ns. Results are presented as means ± SEM. veh = vehicle, ns = not statistically significant.

**Figure 3:**
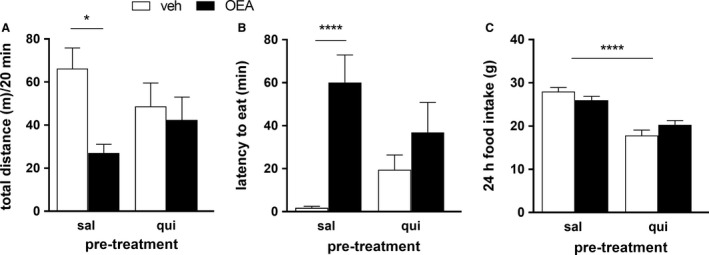
Quinpirole pretreatment blunted the OEA‐induced locomotor inactivity and prolonged latency to eat. All animals (*n* = 5 [A] *n* = 8 [B–C],) were pretreated with Quinpirole (1 mg/kg BW) or saline 1 h prior to OEA (10 mg/kg BW) or veh administration. Total activity in an open field test (A), 2‐way ANOVA, main effect of treatment (veh/OEA) *F*(1,16) = 6.119 *P* < 0.05, pretreatment (sal/qui) *F*(1,16) = 0.01376 ns, treatment × pretreatment *F*(1,16) = 3.204, *P* = 0.09 followed by Bonferroni's post hoc test. Latency to eat (B), 2‐way ANOVA, main effect of treatment (veh/OEA) *F*(1,28) = 14.12, *P* < 0.01, pretreatment (sal/qui) *F*(1,28) = 0.07442 ns, treatment × pretreatment *F*(1,28)  = 4.136, *P* = 0.05 followed by Tukey's post hoc test. 24 h food intake (C), main effect of pretreatment (sal/qui) *F*(1,28) = 61.89, *P* < 0.01, treatment (veh/OEA) *F*(1,28) = 0.058, ns, treatment × pretreatment *F*(1,28) = 4.969, *P* < 0.05, followed by Sidak's post hoc test. Results are presented as means ± SEM. qui, Quinpirole, sal, saline, veh, vehicle, ns, not statistically significant, *P < 0.05, ****P < 0.0001.

**Figure 4:**
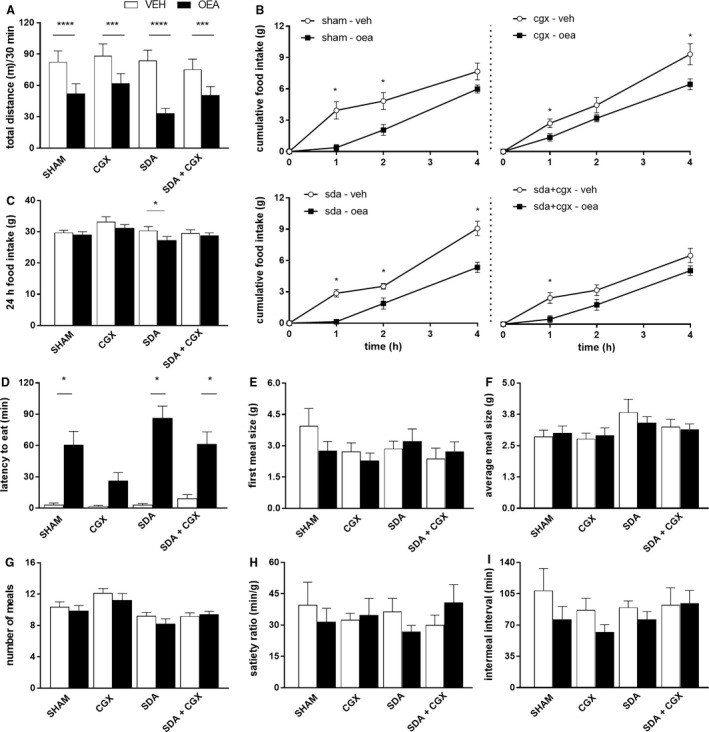
OEA still affected food intake and locomotor activity in CGX, SDA and SDA + CGX animals. (A) locomotor activity measurements in an open field test in Sham, SDA, CGX or SHAM + CGX animals (*n* = 9 per surgery group) after infusion of 10 mg/kg BW OEA or veh, 2‐way ANOVA, treatment *F*(1,36) = 89.86 *P* < 0.01, surgery *F*(3,36) = 0.6705 ns, group × surgery *F*(3,36) = 2.961 *P* < 0.05. (B–I) food intake measurements in Sham, SDA, CGX or SHAM + CGX animals (*n* = 8/9 per surgery group) after administration of 10 mg/kg BW OEA or veh. Short‐term (4 h) food intake (B) sham: 2‐way ANOVA, treatment *F*(1,7) = 13.43 *P* < 0.01, time (3,21) = 92.9 *P* < 0.01, treatment × time *F*(3,21) = 6.047 *P* < 0.01; CGX: treatment *F*(1,7) = 22.57 *P* < 0.01, time *F*(3,21)  = .87 *P* < 0.01, treatment × time *F*(3,21) = 6.091 *P* < 0.01; SDA: treatment *F*(1,32)  = 65.45 *P* < 0.01, time *F*(3,32) = 10.54 *P* < 0.01, treatment × time *F*(3,32) = 10.17 *P* < 0.01; SDA + CGX: treatment *F*(1,31) = 13.54 *P* < 0.01, time *F*(3,31) = 60.44 *P* < 0.01, treatment × time *F*(1,31) = 1.61 ns. 24 h food intake (C), 2‐way ANOVA, treatment *F*(1,30) = 9.979 *P* < 0.01, surgery *F*(3,30) = 2.208 ns, treatment × surgery *F*(3,30) = 12.46 ns. Latency to eat (D), 2‐way ANOVA, treatment *F*(1,30) = 102.8 *P* < 0.01, surgery *F*(3,30) = 4.521 *P* < 0.01, treatment × surgery *F*(3,30) = 5.047 *P* < 0.01. First meal size (E), 2‐way ANOVA, treatment *F*(1,30) = 0.4848 ns, surgery *F*(3,30) = 1.05 ns, treatment × surgery *F*(3,30) = 1.222 ns. Average meal size (F), 2‐way ANOVA, treatment *F*(1,30) = 0.01004 ns, surgery *F*(3,30) = 1.984 ns, treatment × surgery *F*(3,30) = 0.532 ns. Number of meals (G), 2‐way ANOVA, treatment *F*(1,31) = 3.678 ns, surgery *F*(3,31) = 6.533 *P* < 0.01, treatment × surgery *F*(3,31) = 0.988 ns. Satiety ratio (H), treatment *F*(1,29) = 0.074 ns, surgery *F*(3,29) = 0.1273 ns, treatment × surgery *F*(3,29) = 1.17 ns. Intermeal interval (I), treatment *F*(1,30) = 5.792 *P* < 0.05, surgery *F*(3,30) = 0.48 ns, treatment × surgery *F*(3,30) = 1.082 ns. When the main effect or interaction terms were significant, Sidak's post hoc analyses were performed. Results are presented as means ± SEM. veh, vehicle; SDA, subdiaphragmatic vagal deafferentation; CGX, celiac superior mesenteric ganglionectomy; ns, not statistically significant, *P < 0.05, ***P < 0.001, ****P < 0.0001.

**Figure 5:**
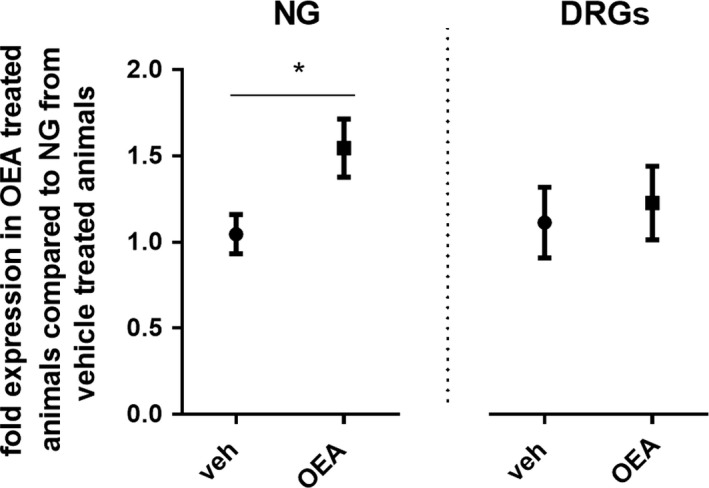
OEA induced the expression of c‐fos m‐RNA in Nodose Ganglia. Relative expression of c‐fos mRNA in NG and DRGs 45 min after veh or OEA (10 mg/kg BW) treatment. (*n* = 7/8). NG: Student *t* test, *P* < 0.05; DRG: Student *t*‐test, ns. Results are presented as means ± SEM. veh, vehicle; NG, nodose ganglia; DRG, dorsal root ganglia; ns, not statistically significant; *P < 0.05.

All statistical analyses were performed using GraphPad prism (v .7.02 for Windows). When data were normally distributed (Shapiro–Wilk test), outliers were detected, using the Rout test. Differences were analyzed using Student *t* test for unpaired normally distributed values of equal variance (Figs. 1J–K, 2A, C, E–I and 5) or a Mann–Whitney *U* test for unpaired comparison of nonnormally distributed data (Fig. 2D). For samples/groups >2, differences were analyzed by a one‐way ANOVA if normality criteria were met (Fig. 1B, D–I), otherwise by the Kruskal–Wallis test (Fig. 1C). Multiple comparisons were assessed with Dunn's test. Where the dependent variable was affected by two factors, data were analyzed with a two‐way ANOVA, (Figs. 1A,L, 2B, 3 and 4). For post hoc analyses, the Bonferroni/Sidak correction was used. Data are presented as means ± SEM. *P* values <0.05 were considered significant.

## Results

### Exogenously administered OEA concurrently reduced food intake and locomotor activity but did not induce avoidance

In line with previous findings (Rodriguez de Fonseca et al. 2001; Fu et al. 2003; Gaetani et al. 2003), 5 and 10 mg/kg BW OEA reduced food intake of freely eating rats compared to vehicle, 1 and 4 h after IP infusion, whereas 1 mg/kg OEA reduced food intake only at 4 h after injection (Fig. 1A). Food intake of all OEA infused animals was similar to food intake of control animals at 8, 12 and 24 h after infusion (Fig. 1B). Five and 10, but not 1 mg/kg BW OEA prolonged the latency of eating onset after infusion compared to controls (48.5 ± 15 and 63 ± 7 min vs. 2 ± 1 min, mean ± SEM) (Fig. 1C). Five and 10 mg/kg OEA did not affect first meal size, average meal size, number of meals, intermeal intervals nor the satiety ratio compared to controls, but 1 mg/kg OEA affected satiety ratio and intermeal interval. We then assessed the effects of OEA on the animals’ locomotor behavior by measuring their activity for 30 min in an Open Field arena. This test revealed that both doses of 5 or 10 mg/kg of OEA reduced the total distance moved compared to vehicle (Fig. 1I). Furthermore, OEA elicited abnormal motor behaviors, here defined as “abdominal writhes” and “lateral torsions”, absent in control animals (Fig. 1J–K) (Video S1) and without, as described previously (Rodriguez de Fonseca et al. 2001; Proulx et al. 2005), inducing conditioned avoidance (Fig. 1L).

### Delaying food access for 1 h after injection prevented OEA's effects on food intake and locomotion

To test whether the prolonged latency to eat and, hence, the reduced food intake in response to OEA might be related to the impaired locomotion, we evaluated the effects of OEA on food intake when animals did not have access to food for the first hour after infusion, once locomotion was no longer impaired. We first verified that one hour after OEA infusion the animals’ motility was no longer affected (Fig. 2A) and then analyzed their eating behavior. Under these conditions, 10 mg/kg OEA did not affect food intake compared to vehicle infusion (Fig. 2B and C), and no difference in latency to eat (Fig. 2D) or eating patterns (Fig. 2E and I) was observed. This indicates that the anorectic effect of IP OEA (10 mg/kg BW) does not persist after locomotor impairments have stopped. It suggests a possible causal relation between impaired locomotion and latency to eat.

### Pretreatment with the dopamine D2/D3 receptor agonist Quinpirole antagonized the locomotor impairment and the delay in eating onset caused by OEA

To examine further whether the delayed eating onset might be causally related to the compromised locomotion, we pretreated the rats with the dopamine D2/D3 receptor agonist Quinpirole in an attempt to prevent the OEA‐induced inhibition of locomotion. Quinpirole prevented OEA from causing a significant reduction in horizontal activity compared to vehicle: while OEA significantly affected locomotion in saline‐pretreated rats, it failed to do so in rats infused with Quinpirole (Fig. 3A). Likewise, OEA again prolonged the latency to eat in control animals, but not in Quinpirole‐treated animals (Fig. 3B). In line with other findings (Kuo 2001), Quinpirole did, however, reduce 24 h food intake compared to saline injected controls, independent of the OEA treatment (Fig. 3C).

### Neither subdiaphragmatic vagal deafferentation nor celiac superior mesenteric ganglionectomy or the combination of both procedures eliminated the motor impairment or reduction in food intake by OEA

To shed further light on the route engaged in IP OEA's signaling to the brain, we first determined whether the OEA‐induced motor dysfunctions were still present in SDA animals and then evaluated the potential involvement of spinal afferents. To do so, we used the established SDA and CGX models and assessed IP OEA's effects on food intake and locomotion. Infusion of 10 mg/kg BW OEA resulted in a comparable reduction in locomotion in all surgery groups (Fig. 4A). OEA also still affected short‐term food intake to different degrees and at various time points in all groups (Fig. 4B) including the 24 h food intake in the SDA group (Fig. 4C). The delayed onset of eating was still present in Sham, SDA and SDA + CGX animals, but did not reach significance in CGX animals (Fig. 4D). Again, OEA had no effect on the animals’ eating patterns (first meal size, average meal size, number of meals, intermeal interval or satiety ratio), independent of the surgical procedure (Fig. 4E–I). Similar to the findings with OEA, the exogenous PPAR*α* agonist WY‐14643 affected the latency to eat and short‐term food intake in all surgical groups without altering other meal pattern parameters (data not shown).

### OEA induced an increase in c‐Fos mRNA in the nodose ganglia but not in dorsal root ganglia

To investigate further whether spinal and/or vagal fibers are recruited for OEA's signaling, although they are not required for the locomotor impairments, we quantified by RT‐qPCR the mRNA levels of c‐*Fos*, a marker of neuronal activation, in NGs and DRGs 45 min after IP OEA infusion. This analysis revealed an upregulation of c‐*Fos* mRNA in NGs of OEA‐treated animals compared to vehicle (Fig. 5). No difference was detectable in DRGs.

## Discussion

This study aimed at extending our understanding of intraperitoneal‐OEA (IP OEA)'s anorectic effect by: (1) investigating, so far largely ignored, locomotor side effects of IP OEA that might influence its anorectic action, and (2) exploring possible peripheral neural pathways for IP OEA's signaling to the central nervous system. We show that the commonly used anorectic dose of IP OEA (10 mg/kg) impaired locomotion and evoked abnormal motor behaviors that are concurrent to OEA's anorectic effect. Furthermore, our attempts to isolate OEA‐induced anorexia from the locomotor impairments remained unsuccessful: neither delaying food access until locomotor impairments stopped, nor a pharmacological rescue of the locomotor impairments, dissociated these two phenomena, suggesting that the IP OEA‐induced anorexia is probably due to locomotion impairment. In addition, we show that these effects were unrelated to conditioned avoidance, did not require intact abdominal vagal afferents and were not completely absent in animals whose intestinal splanchnic nerves were transected. Our data indicate that spinal afferents and an impaired locomotion may both contribute to IP OEA‐induced anorexia.

IP OEA has been shown to reduce food intake in fed and fasted rodents kept on chow or high fat diet (HFD). However, in food‐deprived rats IP OEA also decreased meal size (i.e., caused satiation) (Gaetani et al. 2003; Azari et al. 2014), in ad libitum fed rats, the anorectic effect was almost exclusively due to a prolonged latency to eat (Rodriguez de Fonseca et al. 2001; Gaetani et al. 2003; Azari et al. 2014). We observed similar effects of IP OEA on meal patterns under our conditions: in ad libitum chow‐fed rats, IP OEA selectively prolonged the latency to eat, an effect confined to the first 60 min after OEA infusion (IP OEA (10 mg/kg) postponed eating onset by 63 ± 7 min compared to vehicle). Furthermore, we observed that during the time window in which the animals did not approach food, they displayed impaired locomotor activity, reflected by a decrease in total mobility, associated with abnormal motor behaviors such as abdominal writhes and lateral torsions. These observations are in line with previous findings of experiments (Proulx et al. 2005; Wang et al. 2005) in which OEA bought from different sources was used in comparable doses (5–20 mg/kg in rats) and dissolved in a similar way (5% Tween 80, 5% propylene glycol, and 90% physiological saline). Because of the transient nature of the observed abnormal behaviors, it appears possible that several studies that did not describe such behaviors after IP‐OEA simply did not notice these transient phenomena.

Our findings, as well as a series of data collected by other groups (Rodriguez de Fonseca et al. 2001; Proulx et al. 2005), indicate that the IP OEA‐induced anorexia is not due to conditioned avoidance. As such tests are very sensitive to disturbed wellbeing, the lack of conditioned avoidance virtually excludes that IP‐OEA inhibits eating by inducing overall sickness or malaise. It is therefore interesting that IP OEA at anorectic doses impaired locomotion. In addition, if we started to record activity 1‐h post‐IP OEA infusion, we did not detect any locomotion impairment. Likewise, if we prevented access to food until 1 h after infusion, OEA did not affect the latency to eat or cumulative food intake, raising the possibility of a causal relation between impaired locomotion and prolonged latency to eat. To further examine this possibility, we pretreated rats with the dopamine D2/D3 agonist Quinpirole in an attempt to restore motor activity in OEA‐infused animals to a comparable level of vehicle‐infused animals, as Quinpirole was shown to increase motor activity. This was supposed to allow for the examination of OEA's effects on eating in animals with uncompromised locomotion. In our setting, Quinpirole prevented the motor impairment for the first 20 min after IP OEA and attenuated the prolongation of the latency to eat by IP OEA. These findings support the assumption of a causal relationship between hypolocomotion and anorexia. Nonetheless, our attempts to dissociate the impaired locomotion from the anorectic effects (by delaying food access or by pharmacologic intervention with Quinpirole) were imperfect; in one case we may have missed the anorectic effect OEA may have in the first hours after administration, and in the latter we found that Quinpirole itself had an effect on food intake. Although these results do not conclusively answer whether the IP‐OEA‐induced anorectic effect recapitulates physiological satiety, they raise serious questions about the physiological relevance of IP OEA‐induced anorexia.

While we co‐administered OEA with Quinpirole simply in an attempt to restore normal locomotion after OEA administration, a possible involvement of dopamine in the signaling cascade of OEA cannot be ruled out. Several studies have suggested a link between OEA and the dopamine system (Melis et al. 2008; Luchicchi et al. 2010; Tellez et al. 2013; Hankir et al. 2017). The proposed mechanism involves a nongenomic effect of the PPAR*α* receptor activation, which would activate protein kinases responsible for the phosphorylation status of the nicotinic acetylcholine receptors (nAChRs) modifying the response of dopaminergic neurons to nicotine (Melis et al. 2008; Luchicchi et al. 2010). Together, these findings raise the possibility that pharmacologic amounts of IP OEA, through the PPAR*α*‐nAChR‐DA receptor cascade, alter the firing rate of DA, thus inducing the typical OEA‐dependent motor abnormalities, which are prevented when the DA D2/D3 receptor agonist Quinpirole is administered. Further support for this possibility, and in particular for a role of PPAR*α* in this context, is derived from the fact that animals treated with the exogenous PPAR*α* agonist WY‐14643 showed similar eating patterns (Azari et al. 2013) and, based on our observations, appeared to display similar effects on locomotor activity as animals treated with OEA.

As a known agonist of PPAR*α*, OEA's role in modulating lipid metabolism has been extensively characterized. It promotes lipid utilization and catabolism (Guzman et al. 2004), increases ketone body production (Guzman et al. 2004; Azari et al. 2013) and decreases liver triglyceride and cholesterol levels (Fu et al. 2005). Furthermore, OEA stimulates lipid translocation (Fu et al. 2003), and lipid uptake and intracellular transport (Yang et al. 2007). All these findings are in line with the notion that enterocytes produce OEA in response to a fatty meal (mainly upon ingestion of oleic acid) (Artmann et al. 2008). Once intracellular OEA reaches the concentration of 300‐400 *μ*mol/L, it activates the PPAR*α* receptor (Schwartz et al. 2008), one of the key regulators of lipid metabolism. While these findings seem to reflect a physiological effect of OEA, our data question the concept that the inhibition of eating by exogenous OEA recapitulates such a physiological effect. Rather, our findings suggest that the observed decrease in food intake is secondary to the impaired locomotion observed in response to IP OEA at pharmacological doses. Under these conditions, any residual physiological anorectic effect of OEA would be masked by, and difficult to be discriminated from, the pharmacological side effect.

It was reported that OEA's anorectic effect requires vagal afferents (Rodriguez de Fonseca et al. 2001; Fu et al. 2003; Tellez et al. 2013). In support of this idea, OEA activated vagal afferent neurons in cultures of nodose ganglia neurons (Wang et al. 2005). We showed that IP administration of OEA‐induced *c‐Fos* activation in rats’ nodose ganglia in vivo, strengthening the notion that OEA can signal through abdominal vagal fibers. Nevertheless, the OEA‐induced reduction in food intake and the OEA‐induced locomotor incapacitation, both, were still present in our SDA animal model that has no vagal afferents from below the diaphragm left (Norgren and Smith 1994; Arnold et al. 2006). This confirms previous findings from our laboratory (Azari et al. 2014) and indicates that intact abdominal vagal afferents are not necessary for the anorectic effect of IP administered exogenous OEA. Of course this does not exclude the possibility that a potential physiologically relevant metabolic or satiating effect of OEA may be mediated through the vagus nerve. More so as vagal afferent neurons express PPAR*α* (Liu et al. 2014). Yet, the very strong effects of OEA on the latency to eat associated with the locomotor incapacitation do not require intact vagal afferents, are presumably not physiological, and might mask any residual physiological satiating effect that IP OEA may have. Because in SDA rats, IP OEA's anorectic effect was still present we attempted to shed light on OEA's route of action, by investigating the potential involvement of spinal afferent fibers. All our surgical groups (Sham, SDA, CGX, SDA + CGX) displayed the characteristic IP OEA‐induced locomotor impairment, but IP OEA affected their eating behavior to different degrees. The IP OEA‐induced reduction in food intake was still present after 24 h in SDA animals. This suggests that interfering with vagal fibers would enhance the IP‐OEA effect on eating. We have no plausible explanation for this phenomenon and can only speculate that by altering the vagal innervation of the gastrointestinal tract, we may induce some imbalance in the gut‐brain axis and the enteric nervous system that makes these animals more sensitive to the effects of IP OEA. Furthermore, CGX animals treated with OEA showed an attenuated prolongation of the latency to eat. The short‐term effect of IP OEA on cumulative food intake was still present, but also reduced in CGX animals. These data suggest that IP OEA may prolong the latency to eat in part via spinal afferents and in part via the motor incapacitation, which does not seem to require intact splanchnic afferents. We also showed, though, that IP OEA did not lead to an up‐regulation of *c‐Fos* in the DRGs, which argues against an activation of the spinal fibers by OEA. Furthermore, the CGX surgery is not specific for spinal afferents but also damages sympathetic efferents, potentially modifying the normal physiology of the gastrointestinal tract. Thus, further studies should critically examine whether there could be any other, nonspecific effect of CGX that might antagonize the prolongation of the latency to eat by IP OEA. Either way, while a neural route for OEA signaling cannot be excluded, IP OEA may also act on the brain stem by reaching the area postrema through the blood stream. This interpretation would be in line with the increase in c‐Fos in the AP observed after IP OEA (Romano et al. 2017).

A last consideration is that in our set of experiments we focused on the dose of 10 mg/kg as the rat studies that reported an anorectic effect of OEA, suggesting that this effect may be physiologically relevant, employed doses ranging from 5 to 20 mg/kg (Rodriguez de Fonseca et al. 2001; Fu et al. 2003; Gaetani et al. 2003). Yet, the dose of 1 mg/kg OEA, which does not impair locomotion while reducing 4 h food intake and affecting satiety ratio and intermeal interval, could actually be the most appropriate dose to employ in future rat experiments addressing the physiological relevance of exogenous OEA.

In sum, our findings show that IP OEA, at the dose that in our hands as well as in previous studies (Fu et al. 2003; Gaetani et al. 2003; Azari et al. 2014), reliably reduces food intake, causes locomotor incapacitation, which is the likely cause of the observed OEA‐induced anorexia under the conditions tested. The dissociation of this locomotor impairment from the effects on food intake is crucial to address the mechanisms of any physiological eating‐inhibitory effect of this compound.

Our findings are therefore important and require further investigation with respect to mechanistic explanations of the anorectic effect of exogenous OEA, even more so in light of its potential effects on the dopamine system and its cannabinoid‐like nature.

## Perspectives and Significance

Our findings highlight the fact that IP administration of a commonly used dose of OEA transiently but strongly impairs locomotion in addition to inhibiting eating. This indicates that IP OEA does not simply recapitulate the effects of endogenous OEA and, hence, questions the suitability of IP OEA to investigate a putative physiological satiating effect of endogenous OEA. Further, our data question the suitability of using OEA as a pharmacotherapy for weight control. In any case, the effects of IP OEA require further characterization.

## Conflict of Interest

The authors declare no conflict of interest.

## Data Accessibility

## Supporting information




**Video S1:** Effects of intraperitoneally (IP) Oleoylethanolamide (OEA) on the locomotion of rats. The rat on the left panel was infused IP with 10 mg/Kg body weight of OEA just prior to the video recordings. These side effects are confined to the first 45–60 min post OEA infusion.
